# New developments in neurofibromatosis type 2 and vestibular schwannoma

**DOI:** 10.1093/noajnl/vdaa153

**Published:** 2020-11-16

**Authors:** Yin Ren, Divya A Chari, Sasa Vasilijic, D Bradley Welling, Konstantina M Stankovic

**Affiliations:** 1 Department of Surgery, Division of Otolaryngology – Head and Neck Surgery, University of California San Diego School of Medicine, San Diego, California, USA; 2 Department of Otolaryngology Head and Neck Surgery, Harvard Medical School, Boston, Massachusetts, USA; 3 Eaton-Peabody Laboratories and Department of Otolaryngology Head and Neck Surgery, Massachusetts Eye and Ear, Boston, Massachusetts, USA; 4 Speech and Hearing Bioscience and Technology Program, Harvard Medical School, Boston, Massachusetts, USA; 5 Harvard Stem Cell Institute, Harvard University, Cambridge, Massachusetts, USA; 6 Program in Therapeutic Science, Harvard Medical School, Boston, Massachusetts, USA

**Keywords:** acoustic neuroma, clinical trials, drug repositioning, hearing loss, neurofibromatosis type 2, NF2, vestibular schwannoma

## Abstract

Neurofibromatosis type 2 (NF2) is a rare autosomal dominant disorder characterized by the development of multiple nervous system tumors due to mutation in the *NF2* tumor suppressor gene. The hallmark feature of the NF2 syndrome is the development of bilateral vestibular schwannomas (VS). Although there is nearly 100% penetrance by 60 years of age, some patients suffer from a severe form of the disease and develop multiple tumors at an early age, while others are asymptomatic until later in life. Management options for VS include surgery, stereotactic radiation, and observation with serial imaging; however, currently, there are no FDA-approved pharmacotherapies for NF2 or VS. Recent advancements in the molecular biology underlying NF2 have led to a better understanding of the etiology and pathogenesis of VS. These novel signaling pathways may be used to identify targeted therapies for these tumors. This review discusses the clinical features and treatment options for sporadic- and NF2-associated VS, the diagnostic and screening criteria, completed and ongoing clinical trials, quality of life metrics, and opportunities for future research.

Neurofibromatosis type 2 (NF2) is a multiple neoplasia predisposing syndrome characterized by the formation of multiple nonmalignant nervous system tumors throughout the lifetime due to mutation in the *NF2* tumor suppressor gene. The pathognomonic feature of the NF2 syndrome is the development of bilateral vestibular schwannomas (VS)—histologically benign intracranial tumors arising from myelin-forming Schwann cells of the superior or inferior vestibular divisions of the eighth cranial nerve (Figure 1). Most NF2 patients also develop additional cranial, spinal, and peripheral nerve schwannomas, along with meningiomas, ependymomas, and astrocytomas. Ocular and cutaneous manifestations may also occur. Individuals with NF2 exhibit a wide range of phenotypic variability, in part predicted by the type of genetic variant.^1^

**Figure 1.**
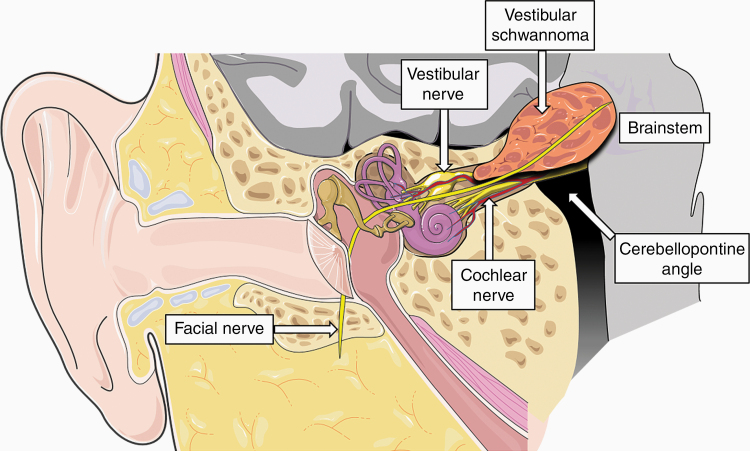
Schematic illustrating vestibular schwannoma arising from a vestibular nerve within the internal auditory canal. The figure was modified from SMART (Servier Medical Art), licensed under a Creative Common Attribution 3.0 Generic License. http://smart.servier.com/.

Treatment of growing NF2-associated and sporadic tumors is driven by severity of symptoms and primarily consists of observation, surgery, or radiation therapy. Currently, there are no FDA-approved pharmacotherapies for NF2 or VS that target the underlying disease pathophysiology. However, burgeoning insights into the molecular biology of sporadic and NF2-associated VS have shed light on the highly variable genotypic–phenotypic correlation of NF2 and suggest numerous putative molecular targets for therapeutic intervention.

In this review, we highlight the clinical features, diagnostic criteria, and treatment options for NF2 and discuss key advances in the treatment of sporadic and NF2-associated VS. We also highlight quality of life (QOL) issues and avenues for future research.

## Epidemiology

VS accounts for 8% of all intracranial tumors with an incidence rate of approximately 1 in 24,000.^2^ While 95% of VS are unilateral, sporadic and typically develop between the fourth and fifth decades of life, bilateral VS occurs in less than 5% of cases and are limited to patients with NF2. The incidence of NF2 is estimated to be around one in 33,000 live births based on a study by Evans et al.^3^ In 1992, the diagnostic prevalence of NF2 was estimated to be about one in 210,000 individuals, but improvements in diagnostic techniques and imaging modalities have led to an apparent increase to one in 100,000 people by 2005.^3,4^ Unfortunately, epidemiological research on NF2 is complicated by de novo mutation rates and rarity of the disease. Moreover, the diagnostic window of NF2 is short between the diagnosis (mean age of 28 years) and death (mean age 39 years).^5^ No prevalence has been described based on ethnicity.

## Pathophysiology

### Genetics of NF2

**Figure 2.**
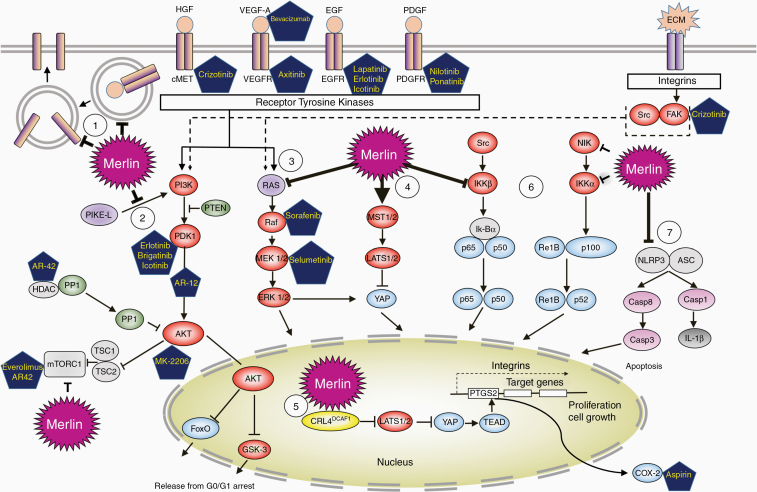
Merlin-regulated signaling pathways and current therapeutic targets in vestibular schwannoma. Merlin protein suppresses cell growth and proliferation by acting at multiple levels in a cell. Details are included in Supplementary Materials.

The *NF2* gene product, merlin, is a cell membrane protein that functions as a tumor suppressor.^5^ Merlin is involved in various signaling pathways including Ras/Raf/MEK/ERK, PI3K/Akt/mTORC1, NF-kB, and Hippo signaling pathways (Figure 2).^6–9^ Mutations in merlin results in changes in its interactions with the actin cytoskeleton.^10,11^ Welling et al. studied a series of patients with VS and found that the frequency, type, and distribution of *NF2* mutations were different between sporadic and familial NF2-associated tumors. Specifically, point mutations in the NF2 gene exons accounted for 58% of mutations in NF2, whereas they only accounted for 19% in unilateral cases. Additionally, small deletions accounted for 76% of the mutations in unilateral VS but only 42% of bilateral NF2-associated tumors. CpG hotspots were identified as predisposing to mutation.^12^

Multiple groups have attempted to determine whether the specific underlying genotype can predict disease severity. Patients with severe clinical disease tend to harbor deletional mutations that produce a truncated or unstable protein that result in complete loss of merlin function, as a result of either nonsense or frameshift mutations in key exons 2 through 13.^1^ By contrast, patients with milder forms of disease tend to contain missense, splice site, or nontruncating mutations, which likely result in some merlin expression.

### Hearing Loss

**Figure 3.**
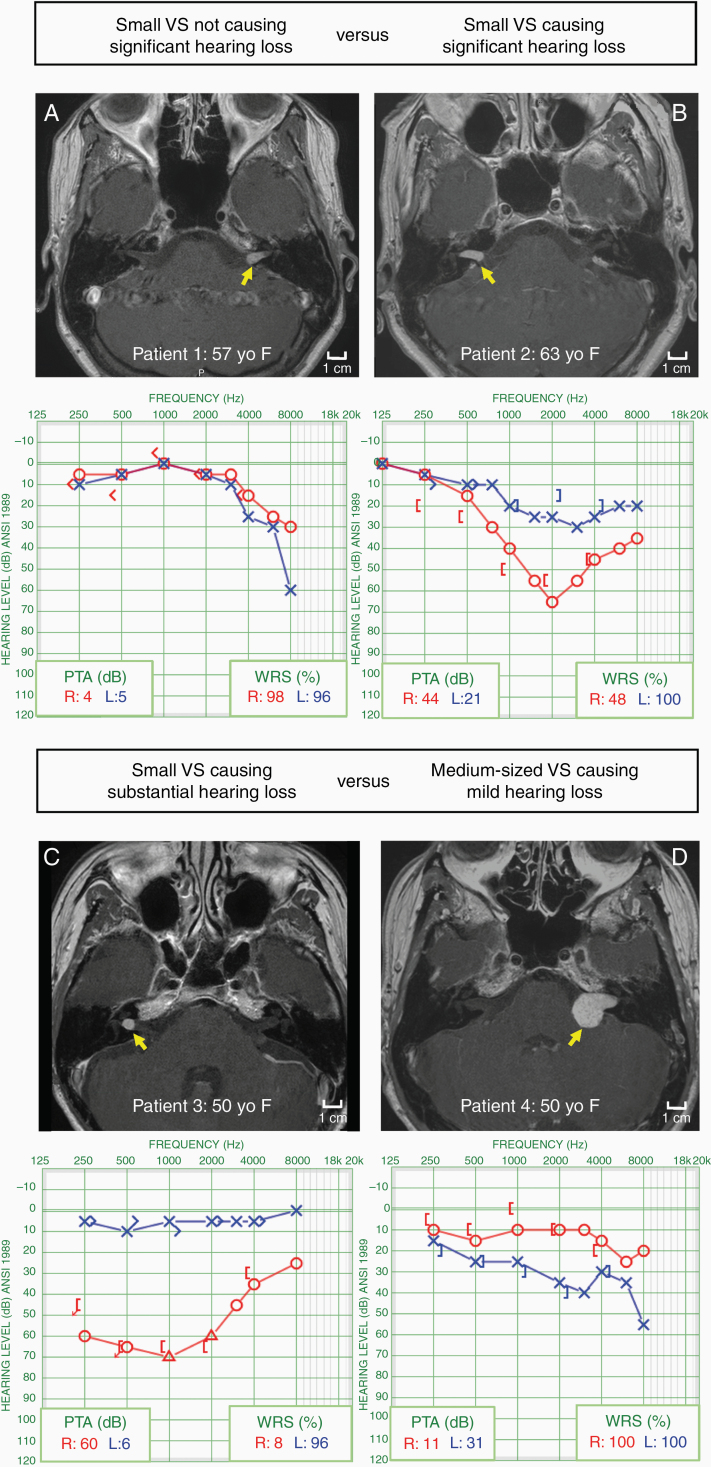
Examples illustrating that vestibular schwannoma (VS) size does not correlate with hearing loss. PTA, pure tone average. Yellow arrow points to VS in all scans. Details are included in Supplementary Materials.

The mechanism by which hearing loss occurs in sporadic and NF2-associated VS has been intensely studied yet still remains incompletely understood. Cochlear nerve dysfunction is far more common clinically and affects up to 95% of VS patients, whereas only up to 50% of VS patients develop imbalance symptoms.^13^ An intuitive hypothesis suggests that sensorineural hearing loss (SNHL) is caused, at least in part, by tumor-mediated mechanical compression. This theory is based on anatomical evidence that any tumor growth within a tightly confined bony internal auditory canal would result in mechanical stress on the nerve and lead to conduction blockade. However, several studies have demonstrated the lack of a consistent correlation between overall tumor size or intracanalicular configuration and the degree of SNHL (Figure 3).^11^ Additionally, a subset of patients develop progressive audiometric threshold shifts without changes in tumor size.^14^ Finally, sudden hearing loss occurs in up to 20% of VS patients without apparent alterations in tumor configuration.

A second hypothesized mechanism is due to ischemia to the hearing apparatus through the disruption of the cochlear blood supply.^15^ Animal models of vascular disturbances to the inner ear have demonstrated that the cochlea can be exquisitely susceptible to ischemic injury. A retrospective series examined the presence of vascular insult in over 270 patients with VS.^16^ There was a significant correlation between the presence of intratumoral microhemorrhage or fibrosis and poor hearing status. Furthermore, the occurrence of sudden hemorrhagic events within the tumor may result in a rapid increase in pressure and leads to compression of the blood supply.^17^ Nonetheless, such histological changes only occur in a small fraction of the patients and do not entirely account for clinical findings in the cohort.

As we begin to discover inherent genetic differences between VS associated with poor hearing and those with good hearing, an emerging hypothesis proposes that tumor-secreted molecules could directly lead to cochlear damage. Ototoxic and neurotoxic metabolites have been shown to be present directly in tumor secretions and can result in cochlear cellular damage.^18^ Decreased distortion product otoacoustic emissions are commonly observed in VS patients with mild SNHL, suggesting that outer hair cell dysfunction could be a primary event in early hearing loss. Furthermore, postmortem studies of temporal bones with untreated VS also demonstrated that a substantial portion showed cochlear atrophy and organ of Corti degeneration. The surprising reversal of SNHL in 50% of patients treated with bevacizumab might also indicate a therapeutic blockade of neurotoxic or ototoxic effects on the neural and/or inner ear tissues.^19^ In addition, extracellular vesicles secreted by tumor cells, known as exosomes, have also been implicated in mediating cochlear damage.^20^ Collectively, these data provide additional insights into the mechanism by which the ototoxic and neurotoxic components within VS secretions could induce hearing loss.

## Diagnosis and Screening

NF2 is subclassified into 3 groups based on clinical presentation and severity of disease: (1) Wishart; (2) Gardner; and (3) mosaic (segmental).^3^ The Wishart subtype has the most severe clinical presentation with typical onset in the late teens or early 20s; patients typically present with spinal tumors in addition to bilateral VS. The Gardner subtype has a less severe presentation and later onset. While patients present with bilateral VS, the incidence of associated intracranial tumors is lower. Finally, the mosaic, or segmental, NF2 subtype describes a condition in which a mutation occurs in embryogenesis rather than in the germline DNA; therefore, only a portion of the patients’ cells carry the mutation.^21^

### Diagnostic Criteria for NF2

While a number of diagnostic criteria for NF2 have been proposed, the Manchester criteria (2005) have become the most widely used.^22^ A definite diagnosis is made by (1) the presence of bilateral VS or (2) a history of a first-degree family relative with NF2 and the development of either a unilateral VS or least 2 of the following conditions known to be associated with NF2: meningioma, glioma, neurofibroma, schwannoma, or posterior subcapsular lenticular opacities. These criteria also included patients with no family history who have multiple schwannomas and/or meningiomas, but without bilateral VS. Recently, Evans et al. integrated large databases of over 2700 individuals with molecular testing for NF2.^23^ The authors suggested that the terms “glioma” and “neurofibroma” be removed and replaced by “ependymoma” and that testing of the gene *LZTR1* be recommended for individuals with unilateral VS given clinical overlap between *LZTR1*-associated schwannomatosis and NF2. The most current and revised Manchester criteria (2017) for NF2 reflect these changes (Table 1).

**Table 1.** Current and Revised Manchester Criteria for Neurofibromatosis Type 2 (NF2)

1. Bilateral VS, diagnosed before age 70
2. First-degree relative family history of NF2 and unilateral VS, diagnosed before age 70
3. First-degree relative family history of NF2 or unilateral VS or 2 of: meningioma, cataract, schwannoma, cerebral calcification (if unilateral VS and > 2 nonintradermal schwannomas, needs negative LZTR1 genetic testing)
4. Multiple meningiomas (2 or more) and 2 of: unilateral VS, cataract, ependymoma, schwannoma, cerebral calcification
5. Constitutional or mosaic pathogenic NF2 gene mutation in blood or identical mutations in 2 distinct tumors.

### Screening and Monitoring

Ocular abnormalities (retinal hamartomas, epiretinal membranes, and subcapsular cataracts) are recorded in 40% to over 70% of children with NF2.^21,24^ These ophthalmologic manifestations are considered early clues of NF2,^21^ though some children present with neurologic symptoms from intracranial tumors. Mean age at first presentation of NF2 in children varies between 5.5 and 7.0 years of age, while mean age of diagnosis varies from 8.8 to 14.9 years. Tumor burden and associated morbidity and mortality among children are typically higher than the adult NF2 population.^25^ Therefore, children with a family history of NF2 should undergo MRI by 8 years of age and molecular genetic testing to identify *NF2* mutations.

Adults with NF2 require education about potential disease complications and routine ophthalmologic and neurologic assessment. Annual examinations by clinicians familiar with NF2 are suggested, along with management by expert multidisciplinary teams for lifelong surveillance and care. MRI of the brain should be performed at baseline and annually. In certain cases, imaging is needed at more frequent intervals for individuals at high risk for hydrocephalus or brainstem compression. MRI of the spine with contrast should be performed given the predisposition to spinal lesions, but the frequency of this imaging depends on tumor burden and clinical exam findings.^26^

### Biomarker Development

To date, no biomarker-driven trial for NF2 has been performed. Existing clinical factors such as tumor size, growth rate, or radiographic features have all been investigated as potential markers; however, many of them suffer from poor sensitivity and specificity and do not yield consistent results. Two histological indices of cellular proliferation, Ki-67 and MIB-2, have both been retrospectively validated in patients with VS and found to correlate with either tumor growth or recurrence after subtotal resections.^27^ While these findings provide a quantitative assessment of tumor growth at the cellular level, histologic biomarkers can be obtained only after tumor extirpation and thus cannot be utilized for disease prognosis.

Since most nongrowing or slow-growing VS are followed with serial MRI scans, several studies have also investigated the utility of radiographic factors. In one of the largest retrospective series of 31 NF2 patients, neither baseline tumor volume nor growth rate correlated with either radiographic or hearing response to bevacizumab treatment. The only significant biomarker was mean apparent diffusion coefficient, a radiographic measure of tissue edema that may be related to the “leakiness” of tumor blood vessels, which showed a modest correlation with reduction in tumor volume.^28^ A recent study investigated the link between tumor inflammation, vascular permeability, and growth of VS using a tracer for positron-emission tomography and dynamic contracted enhanced MRI. Growing sporadic tumors displayed elevated signals derived from these imaging biomarkers, likely due to increased presence of tumor-associated macrophages.^29^ Findings from these studies motivate further validation in larger prospective cohorts with longer follow-up.

VEGF is a key mediator of angiogenesis and is expressed in nearly all VS. Histological data suggests schwannomas overexpress VEGF, which lead to increased vessel density and abnormal cellular proliferation.^30^ In a murine VS xenograft model, pharmacologic inhibition of VEGF led to anatomic and functional normalization of the vascular architecture within the tumor.^30^ Based on the animal data, a retrospective study was conducted where 31 patients with NF2-asssociated VS and hearing loss were treated with bevacizumab.^28^ Hearing improvements were observed in over half of the patients; however, the therapeutic benefit required long-term dosing, which can be associated with chronic systemic toxicity. Studies to reduce the side effects of bevacizumab such as hypertension and proteinuria are ongoing with modification of dosing regimens. Initial induction doses with reduced maintenance dosing are also being evaluated.

The level of VEGF circulating in the peripheral blood may also function as a serum biomarker to predict which individuals will most likely benefit from anti-VEGF therapy. In a prospective trial of 14 patients with NF2 and ipsilateral progressive hearing loss treated with bevacizumab for 12 months, 36% experienced some hearing improvement.^31^ Elevated blood levels of VEGF-D and stromal cell-derived factor 1⍺ correlated with reduction of tumor size on MRI. Interestingly, patients whose hearing did not improve tended to have higher plasma levels of hepatocyte growth factor (HGF), suggesting that HGF/cMET signaling may play a role in hearing loss and affect the response of VS to bevacizumab. Indeed, recent studies have begun to elucidate the role of cMET signaling in VS.^32^

Tumors of the central nervous system secrete molecules that are typically transported systemically via the circulation of CSF, which represents a source for potential biomarkers.^32^ In a study of 43 CSF specimens obtained from patients with sporadic VS, nearly 100 unique proteins involved in various signaling pathways were found to be dysregulated depending on the size of the tumor.^33^ Specifically, ATP binding cassette subfamily A member 3 (ABCA3) and kruppel like factor 11 (KLF11) were positively correlated with VS tumor size, whereas brain abundant membrane attached signal protein 1 (BASP1) and peroxiredoxin 2 (PRDX2) levels were downregulated as tumors grew. While this study did not examine NF2-associated tumors, these biomarkers should be further validated in prospective cohorts and in NF2 patients.

## Management

### Overview

In the past several decades, there has been an apparent increase in the incidence of VS, presumably attributed to a rise in the detection of incidental tumors with improved imaging techniques. However, there is a decrease in the number of patients undergoing surgical resection for all types of VS, with a proportionally larger decrease in the excision rate of sporadic VS compared to NF2-associated VS between 1997 and 2011.^34^ National data for current management of sporadic VS show an increase in the number of cases treated with conservative “watchful waiting” management including observation and surveillance imaging.^35^

Treatment recommendations for VS are made based on a combination of factors including patient age, tumor size, residual hearing and existing comorbidities. In general, surgery is recommended for patients with younger age and larger tumors, while observation or stereotactic radiation is recommended for patients with increasing age and smaller or asymptomatic tumors. Priorities of treatment involve life-saving measures including relieving brainstem compression or hydrocephalus, followed by preserving facial and auditory function. Patients with NF2 or VS in an only hearing ear present unique treatment challenges as the patient may be faced with bilateral deafness. Hearing rehabilitation following VS removal can be achieved in several ways. Auditory brainstem implants, which provide direct electrical stimulation of auditory neurons in the cochlear nucleus, remain an option when the cochlear nerve requires sacrifice. However, when the cochlear nerve is intact, cochlear implantation (CI) could directly stimulate the cochlear nerve. To date, the majority of studies reporting CI outcomes following resection of VS are focused on NF2 patients with bilateral deafness. Recently, however, the indications for CI have been expanded to include patients with single-sided deafness, thus raising the possibility of CI in patients with unilateral VS. A systematic review comparing 15 studies found that the mean speech discrimination score improved from 30.0% preoperatively to 56.4% following implantation with the majority of patients reporting improvement in tinnitus.

The Synodos for NF2 Consortium, established in 2014 and completed in 2018, attempted to identify novel therapeutic agents for treatment in NF2 tumors. An open access (www.synapse.org/Synodos/NF2) database was created for community sharing and mining of drug treatment. Future treatment may involve combination therapies in which 2 drugs act synergistically to reduce the dose of each drug and thereby limit toxicity.^36^ For example, combination therapy with mTOR kinase inhibitor and dasatinib inhibits proliferation of both human VS cells^36^ and human meningioma cells. Importantly, combination therapy can reduce the dose of each drug and thereby its toxicity if drugs act synergistically. Another approach to combination therapy is concurrent administration of drug and radiation to improve efficacy and reduce toxicity. For example, crizotinib, a small-molecule dual inhibitor of the c-Met and ALK receptor tyrosine kinase, can enhance schwannoma radiosensitivity in the mouse model, reducing the overall dose of radiation required to control tumor growth.^37^

### NF2 Drug Discovery

The traditional drug discovery pipeline suffers from several drawbacks including a lengthy duration ranging from 9 to 12 years, prohibitive costs, and a high failure rate where nearly 9 out of 10 drugs ultimately do not reach the market. By contrast, the computational repositioning of existing drugs already approved by the FDA, where interactions between genes or gene networks and drugs guide the repurposing of such drugs toward new indications, offers a novel and transformative approach toward therapeutic discovery. Drug repositioning takes less time, offers substantial savings in preclinical and phase I/II studies, and typically has a lower failure rate since the safety profiles are largely already known. Historically, drug repositioning has been achieved by either opportunistic or serendipitous approaches. Building on these successes, a systematic approach to reposition therapeutics through leveraging large-scale genomic information could lead to the identification of safe drugs for other indications, especially in diseases such as VS, where no approved pharmacotherapies exist.

To identify FDA-approved drugs with potential for repurposing in VS, Sagers et al. conducted a meta-analysis of human VS transcriptomes and applied gene expressions to a computational drug repositioning algorithm to match with known drug–gene interactions.^38^ Mifepristone (RU486), a progesterone and glucocorticoid receptor antagonist already approved for medical abortion, was chosen as the most promising candidate drug. In a preclinical study, mifepristone reduced cellular proliferation and promoted cytotoxicity in primary human VS cultures regardless of *NF2* mutation, with no apparent adverse effects in Schwann cells. A phase II clinical trial on mifepristone in VS is currently being planned.

Among the list of dysregulated genes in VS, neuroinflammation-related signaling was one of the highest ranked pathways.^38^ The activation of the NLRP3 inflammasome, a multi-protein complex that activates caspase-1 resulting in the production of inflammatory cytokines, has become the subject of growing interest due to its emerging role in inner ear biology. Evidence suggests that NLRP3 mutation is associated with cochlear autoinflammation in conjunction with DFNA34-mediated hearing loss and age-rated hearing loss. Activation of NLRP3 triggers the production of IL-1β, a potent proinflammatory cytokine; treatment with a recombinant human IL-1 receptor antagonist, reversed the hearing loss seen in a family with sensorineural hearing loss and NLRP3 mutations.^39^ In VS, genes associated with NLRP3 were significantly upregulated in patients with poor hearing.^40^ Future work is needed to ascertain the therapeutic role of IL-1β blockade in patients with hearing loss secondary to sporadic or NF2-associated VS.

### Clinical Trials for NF2

Improved understanding of the molecular mechanisms driving NF2 pathogenesis has led to the identification of several potential therapeutic targets (Table 2). According to the type of receptors, signaling pathways and effector molecules they target, current FDA-approved drugs used in clinical trials for NF2 can be arranged as follows: (1) ligand-targeting drugs, (2) RTK-targeting drugs, (3) signaling pathway-targeting drugs, and (4) proinflammatory mediators-targeting drugs (Figure 2).

**Table 2.** Active and Recently Completed Prospective Clinical Trials for NF2-Associated VS (http://clinicaltrials/gov)

Drug	Mechanism of Action	Trial Design	Status
Bevacizumab (Avastin^TM^)	Anti-VEGF monoclonal antibody	Phase II, Multicenter (Johns Hopkins University, National Cancer Institute, Massachusetts General Hospital), USA	Completed, efficacious^33^ *N* = 14; Hearing improvement in 36%, tumor response in 43% [NCT01207687]
		Phase II, Multicenter (Children’s Hospital in Los Angeles, Children’s National Medical Center, Children’s HealthCare of Atlanta, University of Chicago, Indiana University, National Cancer Institute, Children’s Hospital Boston and Massachusetts General Hospital, Washington University – St. Louis, New York University Medical Center, Cincinnati Children’s Hospital Medical Center, Children’s Hospital of Philadelphia, University of Utah), USA	Active, not recruiting *N* = 22 [NCT01767792]
		Phase II, Multicenter (Northwestern University, Dana-Farber Cancer Institute, University of Virginia, University of Washington), USA	Active, not recruiting *N* = 50 [NCT01125046]
Axitinib (Inlyta^TM^)	VEGFR1/2/3 inhibitor	Phase II, Multicenter, New York University, USA	Active, not recruiting *N* = 12 [NCT02129647]
Endostatin	VEGF expression inhibitor	Phase II, Beijing Tiantan Hospital, China	Complete, outcome not reported *N* = 20 [NCT02104323]
Aspirin	Cox 2 inhibitor	Phase II, Multicenter (Massachusetts Eye and Ear, Stanford University, Mayo Clinic, University of Iowa, University of Utah), USA	Active, recruiting *N* = 300 [NCT03079999]
Everolimus	Inhibits mTORC1	Phase II, Hopital Beaujon, France	Completed, efficacious^41^ *N* = 10; Hearing stable, tumor response in 55% [NCT01490476]
		Early Phase I, New York University, USA	Active, not recruiting *N* = 5 [NCT01880749]
		Phase II, University of California Los Angeles, USA	Active, not recruiting *N* = 4 [NCT01345136]
		Phase II, New York University, USA	Completed, ineffective^42^ [NCT01419639]
AR-42	Histone deacetylase inhibitor	Early Phase 1, Multicenter (Stanford University, Johns Hopkins University, Massachusetts Eye and Ear, Mayo Clinic), USA	Active, not recruiting *N* = 5 [NCT02282917]
Crizotinib	FAK1 inhibitor	Phase II, University of Alabama, USA	Active, not recruiting *N* = 19 [NCT04283669]
Selumetinib	MEK1/2 inhibitor	Phase II, Cincinnati Children’s Hospital Medical Center, USA	Active, recruiting *N* = 34 [NCT03095248]
Lapatinib	EGFR/ ErbB2 inhibitor	Early Phase I, Multicenter (House Research Institute, Johns Hopkins Hospital, Massachusetts General Hospital, Washington University Medical Center, New York University, Weil Cornell Medical College, Presbyterian Hospital, Ohio State University Medical Center), USA	Completed *N* = 26 [NCT00863122]
		Phase II, New York, University, USA	Completed, efficacious^43^ *N* = 17; Hearing improvement in 30.8%, tumor response in 23.5% (15% decline in tumor volume) [NCT00973739]
Icotinib	EGFR inhibitor	Phase II, Beijing Tiantan Hospital, China	Unknown *N* = 20 [NCT02934256]
Nilotinib (Tasigna^TM^)	Bcr-Abl inhibitor	Phase II, Toronto Western Hospital, University Health Network, Canada	Terminated *N* = 2 [NCT01201538]

Clinical trials that demonstrated efficacy are shown in green, trials that are ongoing or whose results have not been published yet are shown in yellow, and trials that were reported as ineffective or were terminated are shown in red.

#### Ligand-targeting drugs

Targeting angiogenesis of growing VS with the humanized VEGF monoclonal antibody, bevacizumab, has been the most successful pharmacologic intervention to date. Initial studies indicated that treatment with bevacizumab is effective in 50% of patients, both in terms of hearing improvement and tumor shrinkage on imaging.^19,44^ Further prospective studies, however, revealed that sustained responses could only be achieved with continued treatment, which poses a challenge due to dose-limiting long-term toxicities of the drug.^28^ Studies to reduce the side effects of bevacizumab such as hypertension and proteinuria are ongoing with modification of dosing regimens. Initial induction doses with reduced maintenance dosing are also being evaluated.

#### RTK-targeting drugs

The ErbB family of receptor tyrosine kinases (RTKs) comprises the epidermal growth factor receptor (EGFR)/HER-1, HER-2, HER-3 and HER-4, which are known to play a role in Schwann cell differentiation and proliferation. Several different molecules within these interrelated pathways have been targeted in an effort to halt tumor growth or induce regression of disease. Lapatinib, a HER-1/2 inhibitor, has been shown to have beneficial activity in a phase II clinical trial for NF2 patients with growing VS. In this study, 11 of 17 patients showed reduction in tumor size with 4 patients meeting the criteria for clinical volumetric response; hearing response rate was observed in 4 of 13 patients.^45^ Unfortunately, erlotinib, an EGFR/HER-1 antagonist, has not been shown to be efficacious; one review by Plotkin et al. showed no improvement in radiographic or hearing responses in NF2 patients with progressive VS.^46^

#### Signaling pathway-targeting drugs

Promising targets for treatment of VS are predominantly components of PI3K/Akt/mTORC1 and Ras/Raf/MEK/ERK signaling pathways. Recent evidence suggests that rapamycin inhibition of the mammalian target of rapamycin complex 1 (mTORC1) reducing growth of schwannoma cells. Clinical trials of everolimus, a rapamycin analog that acts as a mTOR kinase inhibitor, have shown mixed results. One phase II clinical trial of 10 patients with NF2-associated VS showed that none of the 9 patients with evaluable disease had objective radiographic or hearing responses.^47^ However, another phase II trial had more promising results; of 9 patients studied, 5 showed inhibition of tumor growth that resumed within 3–6 months after treatment discontinuation.^48^ Other targets of this pathway, such as selumetinib, a MEK1/2 inhibitor, are being studied in phase II clinical trials.

#### Proinflammatory mediators-targeting drugs

Cyclooxygenase 2 (COX-2) enzyme and pro-inflammatory transcription factor, nuclear factor-kappa B (NF-κB)^49^ have been shown to be critical modulators of VS proliferation. In NF2, COX-2 was expressed in nearly all VS and its expression level correlated with the degree of cellular proliferation. In cultured sporadic VS cells, the secretion of prostaglandin E2, a potent inflammatory mediator generated by COX-2, correlated with cell proliferation rate, and clinical COX-2 inhibitors prevented VS proliferation in vitro.^50^ A recent microarray study of 1048 VS, including 111 related to NF2, again confirmed the relationship between COX-2 expression and increased tumor proliferation measured by MIB1 expression. To evaluate the therapeutic potential of COX-2 inhibition, several authors have studied the efficacy of anti-inflammatory medications, such as aspirin, in controlling VS growth. A retrospective series of 347 patients with sporadic VS suggested those who took aspirin for unrelated reasons had slower tumor growth^51^; however, recent retrospective studies did not find a consistent correlation between aspirin intake and VS growth. Despite these controversies, guidelines from the Congress of Neurological Surgeons recommended administration of aspirin for VS patients who are undergoing tumor surveillance.^52^ To clarify the therapeutic role of aspirin, a prospective, randomized, placebo-controlled phase II trial of aspirin use in both sporadic and NF2-associated VS is currently underway (ClinicalTrials.gov identifier NCT03079999). The preclinical studies informed the dose of aspirin used in the current clinical trial, which is higher than most people would have taken in the reported retrospective studies.

### Gene Therapy for NF2

Direct modulation of affected genes in specific cell types represents arguably the most powerful therapeutic strategy for NF2. To accomplish functional expression or inhibition of particular genes in specific tissues, the vector must be effectively delivered to all of the affected cells of interest. Delivery platforms typically include viral vectors such as retroviruses, adenoviruses and adeno-associated viruses (AAV), as well as nonviral means including nanoparticles and polymers.^53^

Much of the recent advancements in gene delivery has been in the treatment of inherited retinal dystrophies, genetic hearing loss, and spinal muscular atrophy (SMA1). While equivalent clinical data do not yet exist for treating NF2 and hearing loss, several preclinical animal studies have shown promising results. In particular, direct injection of an AAV serotype 1 vector encoding caspase-1 (ICE) under the Schwann-cell specific promoter, P0, led to regression of schwannomas in a mouse model.^43^ More recently, gene therapy involving direct injection of AAV1 encoding apoptosis-associated speck-like protein, a newly described schwannoma tumor suppressor, in a human xenograft schwannoma model, reduced tumor growth and resolved tumor-associated pain without detectable toxicity.^54^ Larger gene sequences can be successfully transduced using dual-AAV systems in vivo.^55^ Taken together, results from these studies will help better inform the optimal treatment conditions that will maximize the safety and efficiency of viral gene delivery in NF2.

Alternatively, nonviral vectors such as liposomal-, polymeric-, or peptide-based nanoparticles offer an attractive alternative for targeted delivery of molecular therapeutics. Liposomes have been used to deliver genome editing agents to the cochlea of neonatal mice in a model of dominant genetic deafness.^42^ By decorating the nanoparticle surface with a peptide that target Schwann cells, peptide-based nanoparticles have been utilized to deliver genetic materials to primary human vestibular schwannoma cultures in vitro, resulting in decreased tumor cell secretion of an ototoxic inflammatory cytokine.^53^

Challenges still remain in the development of safe, efficient, and clinically translatable approaches for gene/drug delivery. Refinements in technique of injection via the round-window membrane will improve the pharmacokinetics and pharmacodynamics of the injected drug in the inner ear. Differences in the inner ear volumes between rodent models and patients must be taken into account. Future surgical innovations could help better detect electrophysiological changes in the inner ear associated with therapeutic interventions. The incorporation of robotic tools in otologic surgeries could make surgeries more precise and customized to the unique anatomy.^53^

### Immunotherapy

Research in immunotherapy for central nervous system malignancies such as glioblastomas is an area of active investigation. Therapies directed against tumor-associated macrophages that modulate the degree of immunosuppression hold translational potential.^41,56^ While still at an early stage, immunotherapy that specifically targets tumor inflammation may emerge as a new class of NF2 therapeutics. Early data from over 30 years ago have suggested that tumor extracts or serum from VS patients overexpress immunogenic mediators. This was supported by recent evidence suggesting that proinflammatory cytokines such as IL-1β, IL-6, and TNF-α were found to be upregulated in sporadic VS and tumor secretions.^49^

Furthermore, certain fast-growing VS expressed higher levels of M-CSF and IL-34, 2 factors that could regulate chemotaxis of immune cells including tumor-associated macrophages (TAMs).^57^ Additionally, a greater macrophage infiltration was found in growing sporadic VS compared to nongrowing sporadic tumors.^29^ In a series of 10 NF2-associated schwannomas, both high levels of PD-L1 expression and the presence of TAMs and T-lymphocytes were identified in nearly all specimens.^58^ In another study of 44 sporadic tumors undergoing subtotal resection, increased presence of CD163^+^ TAMs and elevated PD-L1 expression were both significantly associated with tumor aggressiveness and poorer disease control.^59^ Together, macrophage biomarkers represent an ongoing active area of research in VS.

## Quality Of Life

Adults with NF2 consistently report reduced QOL across physical, emotional, and mental domains. Patients suffer substantial morbidity related to loss of function, including hearing loss, facial nerve paralysis, visual disturbances, dysphagia, pain, and imbalance. Traditionally, outcomes on clinical trials were measured by tumor growth and neurologic deficit, but QOL is beginning to gain recognition as important secondary outcomes.

The Response Evaluation in Neurofibromatosis and Schwannomatosis (REiNS) International Collaboration was formed to achieve consensus regarding the design of clinical trials and treatments for neurofibromatosis. VS and other NF2-associated tumors are typically benign and thus traditional outcomes used for management of cancerous lesions, such as overall survival or disease-free survival, may not be relevant. Instead, by focusing on functional outcomes, such as the preservation of hearing and/or facial nerve function, collaborators hope to improve the design and comparability of clinical trials. Plotkin et al. proposed consensus recommendations for response evaluation in NF2 clinical trials, specifically for hearing and facial function.^60^ The group endorsed the use of maximum word recognition score as a primary hearing endpoint, with the 95% critical difference for primary hearing outcomes. Patient reported hearing outcome measures have also been recommended.

Few standardized QOL surveys are available for use in NF2 clinical trials, but in recent years, several groups have produced disease-specific, validated questionnaires for the assessment of QOL in NF2 patients.^61,62^ The Neurofibromatosis Two Impact of QOL (NFTI-QOL) is an 8-item questionnaire followed by an open free-response section and was found to correlate well with clinician estimations of disease impact.^62^ Although NF2 results in the development of benign tumors, the impact of psychosocial issues on NF2 patients is equivalent to that observed in patients with malignant disease.^61^

## Outlook

### Biomarker Discovery

There is growing interest in developing technologies to identify molecular biomarkers of hearing loss in sporadic and NF2-associated VS. Unfortunately, nondestructive tissue biopsy of the inner ear is not feasible; while surgical procedures that provide access to the inner ear, such as labyrinthectomy, are nonhearing preserving. Within the cochlea, perilymph fluid percolates the scala tympani and vestibuli and is enriched in proteins secreted by cells in the inner ear. The perilymph proteome of VS was first assembled using liquid chromatography with tandem MS and consisted of 271 unique proteins, including some with putative roles in hearing loss.^63^ Recently, a novel microneedle device was developed to safely sample perilymph fluid through the round window as demonstrated on cadaveric human temporal bones.^64^ Together, these studies highlight the potential for discovering biomarkers of hearing loss from liquid biopsy of human perilymph. Looking forward, through the increased use of “omics” technologies such as genome-wide screens and high-throughput proteomic and epigenetic studies, additional molecular biomarkers could be discovered and validated in an efficient and unbiased fashion.^65^

### Preclinical Models for NF2

NF2 drug development is often hindered by a relative lack of in vitro models that replicate tumor pathophysiology with high fidelity. Only one transformed and immortalized VS cell line with HPV E6 and E7 oncogenes (HEI-193) exists currently and represents the platform for many NF2 drug screening studies. This Schwann cell line was originally immortalized from a tumor obtained from a 56-year-old NF2 patient with bilateral VS and harbors a mutation that causes a splicing defect in NF2 to express a merlin isoform.^66^ HEI-193 cells exhibit an aggressive growth phenotype that is distinct from sporadic or NF2 tumors and as such, they may not fully and accurately recapitulate the tumor biology. For future drug screening efforts, more VS cell lines need to be established either through immortalization of primary tumors or generation of induced pluripotent stem cell lines, to fully capture the diverse mutational landscape of NF2 tumors. Several xenograft transplantation and transgenic models of NF2 have been developed over the years with varying degrees of success (Table 3).

**Table 3.** Established Animal Models to Study VS and NF2

Animal Model	Advantage	Disadvantage
Periostin-Cre NF2^flox/flox^ mouse Gelhausen et al., 2015^9^	True transgenic model of NF2 and NF2-associated VS	Long time to develop VS, high cost, difficulty maintaining and obtaining synchronous tumors, no obvious cochlear pathology
HEI-93 cells xenografted into mouse sciatic nerve Gao et al., 2015^10^	Rapid tumor formation, ease of access and measurement of tumor growth	Not anatomically accurate (mice do not develop hearing loss or vestibular dysfunction), limited neurological assessment possible
HEI-93 cells with fluorescent protein and luciferase reporters, xenografted into mouse sciatic nerve Saydam et al., 2011^11^	Rapid tumor formation, ease of access, reporter provided a non-invasive and reliable means to monitor growth in vivo by bioluminescence and to locate tumor cells by fluorescence microscopy	Not anatomically accurate (mice do not develop hearing loss or vestibular dysfunction), limited neurological assessment possible
Mouse *Nf2*^-/-^ or HEI-193 cells xenografted in meninges Gao et al., 2015^10^	Intracranial window present for longitudinal imaging; intravital microscopy of blood vessels	Superficial implantation, cannot assess hearing loss or vestibular dysfunction
Mouse *Nf2*^-/-^ Schwann cells grafted into mouse CPA Chen et al., 2019^12^	Appropriate tumor microenvironment, hearing loss and vestibular dysfunction present, intravital microscopy and ultrasound through intracranial window	Not NF2
Mouse SC4 Schwannoma cell line implanted into auditory-vestibular nerve complex Bonne 2016^13^	Appropriate tumor microenvironment, hearing loss, bioluminescence and MRI monitoring tumor growth	Significant hearing at implantation and long recovery, sacrificed at 21 days
Mouse *Nf2*^-/-^ Schwann cells grafted into CPA of rats Dinh et al., 2018^67^	Appropriate tumor microenvironment, hearing loss and vestibular dysfunction present	Not NF2, no intravital microscopy or ultrasound

The type of animal model, its major advantages, and main drawbacks are listed below. Details are included in Supplementary Materials.

Given the wide array of clinical manifestations of NF2, there is an urgent need to develop robust animal models to recapitulate the histological and biological changes observed in patients. The difficulty in making human inferences from murine models was accentuated in the work of the Synodos collaborative group, which showed distinct kinome response to treatment of human schwannomas from the response of mouse schwannoma a cells. A phenotypically large animal model (eg, porcine or non-human primate) might enable in vivo drug screening efforts and safety studies, increasing confidence in the likelihood of success of in-human trials. However, compared to mouse models, the cost associated with large animal models is substantial and time to tumor development may be prolonged. Furthermore, patients with sporadic VS or meningiomas may also be considered as a surrogate model for NF2 patients.

### Therapeutic Delivery

The successful clinical translation of gene therapy strategies requires the development of reliable methods to access the site of disease. One of the major impediments for NF2 gene therapy is the presence of a complex network of barriers that isolates the inner ear from systemic circulation. Similar to the blood–brain barrier, a blood–labyrinth barrier exists in the inner ear that protects the organ of hearing and contributes to its unique semi immune-privileged status. While intratympanic or transtympanic injections are routinely employed by otolaryngologists in the office for middle-ear application of drugs, the concentration of drug that ultimately reaches the inner ear is extremely low and variable.^68^ Alternatively, the therapeutic can be delivered into the inner ear space via the round window membrane or the oval window.^53^ In the future, a systematic and quantitative framework may be helpful to optimize drug delivery systems for sustained intracochlear or intrathecal delivery of gene therapies for NF2.

### Immunotherapy

A greater appreciation of the dysregulation of the immune system in the evolution of NF2 provides the basis for improved therapeutic strategies and clinical outcomes. In one of the first studies specifically focused on the immune phenotype of NF2, the authors compared the cytokine expression profile of 23 patients with NF2 against those of healthy donors.^69^ Elevated levels of IL-10 and TGF-β, were found in NF2 patients, and lymphocytes from NF2 patients exhibited a slower rate of proliferation and secreted less IFN-γ, both suggestive of an immunosuppressed state. HLA-DR^–^CD33^+^CD11b^+^ myeloid-derived suppressor cells, a population of myeloid cells that could inhibit the antitumoral immune response, were significantly enriched in NF2 patients. These cells infiltrated also into NF2 tumors and decreased after tumor resection. Functionally, HLA-DR^–^CD33^+^CD11b^+^ cells expressed higher levels of iNOS, NOX2, and TGF-β while suppressed CD8^+^ T-cell activity. As evidence for the role of immunosuppressive monocytes in inhibiting antitumoral responses continues to emerge, therapeutic agents directed against this cell population could be re-positioned a novel class of drugs for NF2.

## Supplementary Material

vdaa153_suppl_Supplementary_Material

## Notes

**Authorship Statement.** K.M.S. and D.E.B. conceived and designed the review. All authors performed literature reviews. Y.R., D.C. and K.M.S. wrote the manuscript, with editorial input from all authors. S.V., Y.R. and D.C. prepared figures.
