# Supplementary material for: New developments in neurofibromatosis type 2 and vestibular schwannoma
Source: Neurooncol Adv. 2020 Nov 16;3(1):vdaa153. doi: 10.1093/noajnl/vdaa153 (PMC7881257; doi:10.1093/noajnl/vdaa153)
Supplement: vdaa153_suppl_Supplementary_Material [file vdaa153_suppl_supplementary_material.docx]

**TITLE: New Developments in Neurofibromatosis Type 2 and Vestibular Schwannoma**

**AUTHORS:** Yin Ren, Divya A. Chari, Sasa Vasilijic, D. Bradley Welling, and Konstantina M. Stankovic

**SUPPLEMENTARY MATERIAL**

- **Supplementary Text**
- **Supplementary References**

**Merlin-regulated signaling pathways and current therapeutic targets in vestibular schwannoma.** As shown in Figure 2, at the level of RTK, merlin can negatively influence traffic of internalized RTK^1^ (labeled “1”) and inhibit downstream signaling pathways (labeled “2”, “3”). The PI3K/Akt/mTORC1 and the Ras/Raf/MEK/ERK signaling pathways are triggered by RTK (bold lines) and integrins (bold dotted lines). Downstream effectors of these pathways include AKT, FoxO, GSK-3, TSC2, TORC1, and ERK. Activated AKT and ERK accumulate in the nucleus where they phosphorylate their target substrates leading to cell proliferation and survival. Phosphorylation of FoxO and GSK-3 by AKT is followed by their inactivation and nuclear exclusion resulting in release of the cells from G0/G1-arrest. Merlin has the ability to suppress PI3K/Akt/mTORC1 pathway trough the interaction with PIKE-L by preventing phosphorylation and activating PI3K;^2^ merlin can also directly inhibit mTORC1 (labeled “2”)^3^. Merlin can also prevent activation of the Ras/Raf/MEK/ERK pathway by interacting with RAS and RAS-GAP proteins (labeled “3”)^4^. Furthermore, merlin can regulate the Hippo and the NF-kB signaling pathways whose deregulation is also associated with the pathogenesis of VS. In the Hippo pathway, merlin facilitates phosphorylation of MST1/MST2 complex (labeled “4”) ^5^. Additionally, activated merlin accumulates in the nucleus where it inhibits CRL4^DCAF1^ ubiquitin ligase activity which protects LATS1/2 complex from degradation, enabling it to phosphorylate YAP transcriptional coactivator^6^. Consequently, phosphorylated YAP is unable to interact with TEAD and activates transcription of target genes involved in cell growth, oncogenic transformation, epithelial-mesenchymal transition, as well as genes involved in proinflammatory signaling such as *PTGS2* encoding COX-2 (labeled “5”)^7^. In the NF-kappaB signaling pathway, merlin interferes with activity of NIK and IKKα, and inhibits TNF-α-induced IκB degradation which prevents NF-κB-dependent gene transcription (labeled “6”)^8^. Finally, merlin may exert inhibitory effects on inflammasome by negative regulation of ASC and NLRP3, which in turn downregulate activation of various caspase enzymes (Caspase-1/3/8) causing inhibition of apoptosis and reduction of IL-1β secretion (labeled “7”). Current FDA-approved drugs target signaling networks that are dysregulated due to *NF2* mutation and can be arranged into four main groups: 1. Ligand- targeting drugs (bevacizumab), 2. RTK- targeting drugs (crizotinib, axitinib, papatinib, erlotinib, icotinib, nilotinib, ponatinib), 3. Signaling pathways targeting drugs (Ras/Raf/MEK/ERK pathway- sorafenib, selumetinib; PI3K/Akt/mTORC1 pathway- erlotinib, brigatinib, icotinib, AR-12, AR-42, MK2206, everolimus), and 4. Proinflammatory mediators-targeting drugs (aspirin).

Akt, protein kinase B; ASC, apoptosis-associated speck-like protein containing a CARD; Casp, caspase; cMET, hepatocyte growth factor receptor; COX-2, cyclooxygenase-2; CRL4; cullin-ring E3 ligase 4; ECM, extracellular matrix; EGF, epidermal growth factor; EGFR, epidermal growth factor receptor; ERK, extracellular-signal-regulated kinase; FAK, focal adhesion kinase; FoxO, forkhead box, sub-group O; GSK-3, glycogen synthase kinase-3; HDAC, histone deacetylases; HGF, hepatocyte growth factor; IKKα, Ikappa B kinase α; IKKβ, Ikappa B kinase β; Lats1/2, large tumour suppressor 1/2; MEK, mitogen-activated protein kinase; MST1/2 macrophage stimulating 1/2; mTORC1, mammalian target of rapamycin complex 1; NIK, NF-κB-inducing kinase; NLRP3, NLR Family Pyrin Domain Containing 3; PDGF, platelet-derived growth factor; PDGFR, platelet-derived growth factor receptor; PDK1; 3-phosphoinositide-dependent protein kinase-1; PI3K, phosphatidylinositol 3-kinase; PIKE-L, phosphoinositide 3-kinase enhancer isoform 1;PP1, protein phosphatase-1; PTEN, phosphatase and tensin homologue; PTGS2, prostaglandin-endoperoxide synthase 2; RAF, rapidly accelerated fibrosarcoma (raf kinase, effector of Ras); RAS, rat sarcoma (proto-oncogene, GTPase); RelB, RELB proto-oncogene, NF-kB subunit; Src, Src proto-oncogene, non-receptor tyrosine kinase; TEAD, TEA domain family member; TSC1/2, tuberous sclerosis complex 1/2; VEGF-A, vascular endothelial growth factor A; YAP, yes-associated protein. Red = kinases. Gray = complexes (mTORC1, TSC1/2) and other (ASC, NLRP3, HDAC). Green = Phosphatases (PTEN, PP1). Blue = transcription factors (YAP, FoxO). Purple = GTPases (Ras, PIKE-L). Pink = caspases (Casp 1/3/8). Yellow= ligases. Drugs with putative targets are shown in blue pentagons.

**Lack of correlation between VS size and degree of hearing loss.** As shown in Figure 3A, a 57-year-old (yo) female (F) with a left-sided VS (yellow arrow) discovered incidentally when she suffered a sudden hearing loss in the contralateral right ear and underwent MRI scan. The sudden loss on the right side responded to oral prednisone burst and taper and recovered to normal. Her tumor on the left was non-growing over several years of observation. In Figure 3B, a 63 yo F with a right-sided VS discovered on MRI scan performed for evaluation of a gradual decline in hearing on the right side over several years leading to a poor word recognition score (WRS) of 48%. Her tumor was non-growing over several years of observation. In Figure 3C, a 50 yo F with a small right-sided intracanalicular VS diagnosed on MRI scan preformed for evaluation of gradual decrease in hearing on the right side and poor WRS of 8%. She developed right-sided hemifacial spasm. Finally, in Figure 3D**,** a 50 yo F with a left-sided VS diagnosed after she developed symptoms of vertigo and a mild asymmetry in hearing. Vestibular testing revealed peripheral vestibular hypofunction on the left side. She was treated with oral prednisone burst and taper and one intratympanic injection of dexamethasone without improvement in hearing.

**Animal models for neurofibromatosis type 2 and vestibular schwannomas.** As shown in Table 3, xenograft transplantation and transgenic models have been developed with varying degrees of success. These include HEI-193 ectopically xenografted onto murine sciatic nerves and meninges of mice bearing intracranial windows ^10,11, 29^, NF2 -/- Schwann cells orthotopically grafted in the cerebellopontine angle of mice^12, 13^ and rats^14^, and the periostin-cre NF2^flox/flox^ transgenic mouse model^9^.

**Supplementary References**

1. McClatchey AI, Fehon RG. Merlin and the ERM proteins - regulators of receptor distribution and signaling at the cell cortex. *Trends Cell Biol*. 2009.

2. Rong R, Tang X, Gutmann DH, Ye K. Neurofibromatosis 2 (NF2) tumor suppressor merlin inhibits phosphatidylinositol 3-kinase through binding to PIKE-L. *Proc Natl Acad Sci U S A*. 2004.

3. James MF, Han S, Polizzano C, et al. NF2/Merlin Is a Novel Negative Regulator of mTOR Complex 1, and Activation of mTORC1 Is Associated with Meningioma and Schwannoma Growth. *Mol Cell Biol*. 2009.

4. Cui Y, Groth S, Troutman S, et al. The NF2 tumor suppressor merlin interacts with Ras and RasGAP, which may modulate Ras signaling. *Oncogene*. 2019.

5. Su T, Ludwig MZ, Xu J, Fehon RG. Kibra and Merlin Activate the Hippo Pathway Spatially Distinct from and Independent of Expanded. *Dev Cell*. 2017.

6. Li W, Cooper J, Zhou L, et al. Merlin/NF2 Loss-Driven Tumorigenesis Linked to CRL4DCAF1-Mediated Inhibition of the Hippo Pathway Kinases Lats1 and 2 in the Nucleus. *Cancer Cell*. 2014.

7. Guerrant W, Kota S, Troutman S, et al. Yap mediates tumorigenesis in neurofibromatosis type 2 by promoting cell survival and proliferation through a COX-2-EGFR signaling axis. *Cancer Res*. 2016.

8. Kim JY, Kim H, Jeun S-S, et al. Inhibition of NF-kappaB activation by merlin. *Biochem Biophys Res Commun*. 2002.

9. Gehlhausen JR, Park S-J, Hickox AE, et al. A murine model of neurofibromatosis type 2 that accurately phenocopies human schwannoma formation. *Hum Mol Genet*. 2015;24(1):1-8.

10. Gao X, Zhao Y, Stemmer-Rachamimov AO, et al. Anti-VEGF treatment improves neurological function and augments radiation response in NF2 schwannoma model. *Proc Natl Acad Sci U S A*. 2015;112(47):14676-14681.

11. Saydam O, Ozdener GB, Senol O, et al. A novel imaging-compatible sciatic nerve schwannoma model. *J Neurosci Methods*. 2011;195(1):75-77.

12. Chen J, Landegger LD, Sun Y, et al. A cerebellopontine angle mouse model for the investigation of tumor biology, hearing, and neurological function in NF2-related vestibular schwannoma. *Nat Protoc*. 2019;14(2):541-555.

13. Bonne N-X, Vitte J, Chareyre F, et al. An allograft mouse model for the study of hearing loss secondary to vestibular schwannoma growth. *J Neurooncol*. 2016;129(1):47-56.

14. Dinh CT, Bracho O, Mei C, et al. A Xenograft Model of Vestibular Schwannoma and Hearing Loss. *Otol Neurotol*. 2018;39(5):e362--e369.
